# Enhancing misinformation correction: New variants and a combination of awareness training and counter-speech to mitigate belief perseverance bias

**DOI:** 10.1371/journal.pone.0299139

**Published:** 2024-02-16

**Authors:** Jana Siebert, Johannes Ulrich Siebert

**Affiliations:** 1 Faculty of Arts, Department of Economic and Managerial Studies, Palacky University Olomouc, Olomouc, Czech Republic; 2 Department of Business and Management, Management Center Innsbruck, Innsbruck, Austria; National Research Council (CNR), ITALY

## Abstract

Belief perseverance bias refers to individuals’ tendency to persevere in biased opinions even after the misinformation that initially shaped those opinions has been retracted. This study contributes to research on reducing the negative impact of misinformation by mitigating the belief perseverance bias. The study explores the previously proposed awareness-training and counter-speech debiasing techniques, further developing them by introducing new variants and combining them. We investigate their effectiveness in mitigating the belief perseverance bias after the retraction of misinformation related to a real-life issue in an experiment involving N = 876 individuals, of whom 364 exhibit belief perseverance bias. The effectiveness of the debiasing techniques is assessed by measuring the difference between the baseline opinions before exposure to misinformation and the opinions after exposure to a debiasing technique. Our study confirmed the effectiveness of the awareness-training and counter-speech debiasing techniques in mitigating the belief perseverance bias, finding no discernible differences in the effectiveness between the previously proposed and the new variants. Moreover, we observed that the combination of awareness training and counter-speech is more effective in mitigating the belief perseverance bias than the single debiasing techniques.

## Introduction

A large body of research is dedicated to reducing the negative impact of misinformation [1–3]. Researchers have proposed various prebunking and debunking interventions, investigating their effectiveness in reducing the negative impact of misinformation on individuals. Prebunking interventions encompass up-front warnings, inoculation, and news and media literacy training [4,5], while debunking interventions include post-warnings, awareness training, and refutations [3,4,6].

Various approaches have been employed to assess the effectiveness of prebunking and debunking interventions in reducing the negative impact of misinformation. Some studies aimed at reducing participants’ perceived accuracy or reliability of claims [7–13], while others focused at mitigating the continued influence effect (CIE) of misinformation in the classical CIE paradigm [14–17] or at mitigating the belief perseverance bias (BPB) after the retraction of misinformation in the classical BPB paradigm [4,18–21].

The CIE refers to the phenomenon in which retracted information continues to influence our inferential reasoning [22]. In the classical CIE paradigm [6], participants receive several small pieces of information about a particular unfolding event, one of which is later retracted. When participants are later asked specific open-ended inference questions about the event, CIE makes them refer to the retracted information. It is worth noting that the current research on CIE is moving away from this traditional paradigm by not limiting itself to unfolding events and providing information in small pieces or using open-ended inference questions about the event [23–26]. BPB is defined as the tendency to persevere in beliefs or opinions even after the information on which the beliefs or opinions were based has been discredited [27]. In the BPB paradigm [28], participants receive information related to a specific issue, which is later retracted. When participants are later asked for their opinion on the issue, they provide an opinion biased by the misinformation instead of returning to their initial opinion held before encountering misinformation.

This paper employs the classical BPB paradigm to examine the effectiveness of two previously proposed debiasing techniques, their modifications, and their combination in mitigating BPB after the retraction of misinformation, aiming to reduce the negative impact of misinformation.

### Techniques to mitigate the belief perseverance bias

Several debiasing techniques have been proposed to mitigate BPB. Anderson et al. proposed counter-explanation and inoculation debiasing techniques [28]. The *counter-explanation technique* is a debunking intervention that prompts recipients of misinformation to write causal explanations for an alternative hypothesis. Anderson’s *inoculation technique*, on the other hand, is a prebunking intervention that prompts potential misinformation recipients to create plausible explanations for both (or all) possible hypotheses before reading a particular piece of information. However, it should be noted that Anderson’s inoculation technique is not a typical inoculation intervention based on McGuire’s inoculation theory [29], as it does not include a warning component.

Siebert and Siebert proposed counter-speech and awareness-training debiasing techniques [4]. The *counter-speech technique* is a debunking intervention that refutes misinformation by presenting convincing arguments against specific claims to misinformation recipients. The *awareness-training technique* involves raising misinformation recipients’ awareness of BPB and its potential negative impact on their opinions when exposed to misinformation. They applied awareness training as a debunking intervention but suggested it could also be applied as a prebunking intervention [4].

The counter-speech and awareness-training techniques outperform Anderson’s inoculation and counter-explanation techniques [4]. Specifically, the counter-speech and awareness-training techniques exhibit higher practical applicability for addressing misinformation spread in media than Anderson’s inoculation and counter-explanation techniques. Additionally, they are more effective in mitigating BPB than the counter-explanation technique [4].

This paper explores the awareness-training (AT) and counter-speech (CS) debiasing techniques and further develops them. Specifically, we introduce new variants of these techniques, combine them, and investigate their effectiveness in mitigating BPB. In contrast to Siebert and Siebert [4], who investigated the effectiveness of AT and CS in relation to misinformation concerning a constructed issue, our study focuses on misinformation concerning a real-life issue.

We anticipate that Siebert and Siebert’s findings regarding the effectiveness of the debiasing techniques AT and CS in mitigating BPB after the retraction of misinformation concerning a constructed issue [4] will extend to misinformation concerning a real-life issue investigated in this paper. We, therefore, formulate the following hypotheses.

**H1:** The AT technique mitigates BPB.
**H2:** The CS technique mitigates BPB.
**H3:** The CS technique is more effective in mitigating BPB than the AT technique.

The CS technique, as proposed by Siebert and Siebert [4], involves presenting arguments that support the validity of an opposing (or alternative) claim to the specific misinformation (we denote this variant CS_opp_). However, this variant of the CS technique can only be applied when arguments supporting the validity of an opposing (or alternative) claim are available. Recognizing that such arguments may not always be accessible at the time of misinformation retraction, we introduce another variant of the CS technique that provides arguments supporting the invalidity of the specific claim of misinformation (we denote this variant CS_inv_). This new variant of the CS technique is more versatile than the original variant CS_opp,_ as it does not necessitate the existence of arguments supporting the validity of an alternative claim; any arguments indicating the invalidity of the specific claim are sufficient.

The AT technique, as proposed by Siebert and Siebert [4], illustrates the potential negative impact of BPB using a simple example involving a hypothetical real-life situation (we denote this variant AT_hyp_). Additionally, we introduce another variant of the AT technique that illustrates the potential negative impact of BPB using one of the most damaging medical hoaxes of the last 100 years—the alleged relationship between the measles-mumps-rubella (MMR) vaccination and autism (we denote this variant AT_hoax_).

In this paper, we compare the variants of CS and AT in terms of their effectiveness in mitigating BPB, addressing the following research questions.

**RQ1:** Do the CS_opp_ and CS_inv_ variants of the CS technique differ in their effectiveness in mitigating BPB?
**RQ2:** Do the AT_hyp_ and AT_hoax_ variants of the AT technique differ in their effectiveness in mitigating BPB?

In addition to examining the effectiveness of the AT and CS variants, we explore the effectiveness of their combination. Research on combining different techniques to reduce the negative impact of misinformation has been limited. Vraga et al. [30] combined news literacy tweets (a prebunking intervention) with short-format refutations, finding that while the combination reduced the negative impact of misinformation, the news literacy tweets did not enhance the effectiveness of refutations. In contrast, Hameleers [8] combined a news media literacy message (comprising a warning about the existence of misinformation, guidance on detecting misinformation by looking at the source and type of evidence, and distinguishing external reality from biased media depictions) with a detailed refutation, finding the combination more effective in reducing issue agreement than the news media literacy message or the detailed refutation alone. Assuming that the individual debiasing techniques AT and CS are effective in mitigating BPB (H1 and H2), we hypothesize that combining both techniques (we denote the combination AT+CS) will also be effective in mitigating BPB. We, therefore, formulate the following hypothesis.

**H4:** The combination of the AT and CS techniques mitigates BPB.

Additionally, given the mixed results from the liminted research on combining techniques to reduce the negative impact of misinformation, we pose the following research question:

**RQ3:** Is the combination of the AT and CS techniques more effective in mitigating BPB than the single debiasing techniques?

## Method

### Study design

We employed a web-based pretest-posttest between-subjects experimental design. In this design, participants were randomly assigned to one of six debiasing treatment conditions—namely, debiasing techniques AT_hyp_, AT_hoax_, CS_opp_, CS_inv_, the combination of the debiasing techniques AT+CS, and a control group (CG). The pretest measured participants’ initial opinions, while the posttest measured their opinions after the exposure to the respective debiasing treatment or control condition.

It is worth noting that various experimental designs have been employed to assess the effectiveness of prebunking and debunking interventions in reducing the negative impact of misinformation. Numerous studies have investigated the effectiveness of corrective interventions by comparing opinions, beliefs, attitudes, or perceived accuracy or reliability of claims among individuals exposed to misinformation and its correction versus those exposed solely to misinformation [1,8,30–34]. While this approach has demonstrated the effectiveness of various corrective interventions in reducing the negative impact of misinformation, it falls short of determining the extent to which corrective interventions revert individuals’ opinions back to the baseline before encountering misinformation [2]. To assess the extent to which corrective interventions revert individuals’ opinions back to the baseline, comparing opinions after the exposure to misinformation and a corrective intervention with those held before the exposure to misinformation is essential [2]. This approach to measuring the effectiveness of corrective interventions has already been employed in previous studies [4,14–17,19,20,22,23,28,35], and we also adopted it in this study.

Most studies investigating the extent to which corrective interventions revert individuals’ opinions back to the baseline have employed the *posttest-only control group design* rather than the *pretest-posttest design* [14–17,19,20,22,23,28,35]. In contrast to the posttest-only design, the pretest*-*posttest design offers the advantage of identifying participants whose opinions are influenced by misinformation and who experience BPB after the retraction of misinformation. This design enables the determination of the percentage of participants experiencing BPB and facilitates the assessment of the effectiveness of the debiasing techniques on these individuals. The pretest-posttest design was utilized in the study on mitigating BPB by Siebert and Siebert [4], and we adopted the same design in our study. To track changes in participants’ opinions throughout the experiment, identify those experiencing BPB, and assess the effectiveness of the debiasing techniques, we measured participants’ opinions four times during the experiment (more details provided in Sec: *Dependent variable—opinion on the issue*).

Studies employing a pretest-posttest design to investigate the impact of misinformation and its correction commonly use the same measurement items for the pretest and the posttest [10,34,36–38]. However, the repeated use of identical measurement items within an experiment can lead to challenges such as a *practice effect* or attempts to *maintain consistency*. Our experiment employs different measurement items at each measurement time to address these issues. Moreover, to mitigate the *item order effect*, we employ *random counterbalancing*. Precisely, just like Siebert and Siebert [4], we determine the order of the measurement items for each participant in the experiment randomly without replacement.

### Power analysis

It is important to note that the data collected herein will serve a dual purpose in addition to addressing the primary objectives outlined in this study. Specifically, the dataset generated from this research will be utilized for subsequent analyses in a follow-up study examining the moderating role of initial opinions and the time of forming initial opinions in the processing of misinformation and its correction. This approach maximizes the utility of the gathered data, enabling a more comprehensive exploration of related research questions and contributing to a broader understanding of the impact of misinformation and its correction.

We performed *a priori* power analyses in G*Power to determine the minimum sample size necessary to detect significant effects in this and the subsequent study. For this study, an *a priori* power analysis suggested a minimum sample size of 318 participants experiencing BPB to detect a medium effect size (partial *η*^2^ = .06) with 1−*β* = .95 and *α* = .05 in between-subjects F-tests with six conditions. An *a priori* power analysis for the subsequent study indicated that 284 participants experiencing BPB were needed. As the groups in the subsequent study are formed using observed independent variables, we decided to increase the sample size by 25% to 355 to ensure sufficient participants in each group. Additionally, given that not all individuals experienced BPB, an accordingly higher total sample was needed. Therefore, we regularly monitored the collected data, precisely the number of participants experiencing BPB, and concluded data collection after reaching the total sample of 876 participants, of which 364 participants exhibited BPB (see Sec: *Effectiveness of the debiasing techniques*).

### Participants

Participants were recruited by online survey provider Qualtrics^©^ in the UK between May and August 2021. We decided to focus our study on young adults. Thus, participants had to be between 18 and 35 years old to be included in the study. Furthermore, only participants with good English were eligible for the study.

We collected data from 876 participants. The sample consisted of 439 females and 437 males. The mean age for participants was 27.5 years (SD = 5.2 years). In terms of education, 477 participants attained a university education, 392 attained a high school education, and seven did not finish high school. Regarding employment, 630 participants were employed, 122 were unemployed, and 124 were students.

### Stimuli

All participants were exposed to (mis)information on the real-life issue concerning remote and in-office work. The (mis)information took the form of a one-page blog article that was entirely fabricated. The article summarized the results of an international study comparing remote and in-office work in terms of benefits for companies, suggesting that companies with employees working remotely are more productive and efficient than those with employees working in an office (see the pro-remote-work misinformation stimulus, Appendix C in **S1 Appendix**). The results of the preparatory study (Appendix A in **S1 Appendix**) indicated that the retraction of this misinformation induces BPB, making this misinformation stimulus well-suited for studying the effectiveness of debiasing techniques.

Each participant was randomly assigned to one of six debiasing treatment conditions and exposed to the corresponding debiasing technique (AT_hyp_, AT_hoax_, CS_opp_, CS_inv_, or AT+CS) or the control treatment (CG). The texts for AT_hyp_, AT_hoax_, CS_opp_, and CS_inv_ were approximately half a page in length, while the text of AT+CS was about one full page (see Appendix D in **S1 Appendix**). Participants in the CG responded to 14 questions concerning security habits at work.

The AT_hyp_ and AT_hoax_ techniques introduced BPB as a phenomenon responsible for irrational behavior. They illustrated the potential negative impact of BPB on people’s opinions with specific examples and provided warnings about its pitfalls. Specifically, the AT_hyp_ technique used a simple example concerning a hypothetical real-life situation to illustrate the potential negative impact of BPB, while the AT_hoax_ technique employed the alleged relationship between the measles-mumps-rubella vaccination and autism for illustration.

The CS_opp_ and CS_inv_ techniques challenged the unsubstantiated claim of the retracted article, which asserted that remote work would increase companies’ productivity and efficiency compared to traditional in-office work. The CS_opp_ technique then pointed out the existence of numerous arguments supporting the opposite claim, i.e., that traditional in-office work was increasing companies’ productivity and efficiency compared to remote work. Conversely, the CS_inv_ technique underscored various arguments suggesting that remote work did not increase companies’ productivity and efficiency compared to traditional in-office work. Both techniques then presented three specific arguments supporting the new claim. Notably, unlike the misinformation stimulus, the arguments provided by the CS_opp_ and CS_inv_ techniques were not fabricated. Instead, they were grounded in actual surveys and research results, and each argument included an active link to the specific source supporting it. Furthermore, both techniques encouraged readers to critically engage with the new arguments, inviting them to contemplate and contribute additional arguments supporting the revised claim.

The combination treatment AT+CS comprised the arbitrarily chosen variants AT_hoax_ and CS_opp_ of the respective debiasing techniques. Participants were sequentially exposed to AT_hoax,_ followed by CS_opp_. It is important to note that we deliberately opted for a single combination of AT and CS variants in the study to maintain a reasonably low total number of debiasing treatment groups.

### Dependent variable—opinion on the issue

We measured participants’ opinions on the relationship between the work location (remote vs. in-office work) and companies’ productivity and efficiency (shortly *opinion on the issue*) four times during the study (at measurement times *t*_*1*_: initial opinion, *t*_*2*_: opinion after the exposure to misinformation, *t*_*3*_: opinion after the retraction, *t*_*4*_: opinion after the debiasing treatment). At each measurement time, participants’ opinions on the issue were measured with four Likert items evaluated on a 7-point scale (1 = *completely disagree*, 4 = *neither agree nor disagree*, 7 = *completely agree*). To ensure robust measurement, we developed and validated 16 pairs of oppositely worded Likert items in the preparatory study (see Appendices A and B in **S1 Appendix** for details on the preparatory study and the Likert items, respectively). Examples of these items include statements such as “Companies with employees working remotely tend to perform better than companies with employees working in an office” and “Employees working in an office tend to get more work done than employees working remotely”.

We applied random counterbalancing to mitigate the item order effect in the measurement of participants’ opinions on the issue. This involved randomly selecting, without replacement, Likert items from the set of 16 pairs for each participant at each measurement time. Additionally, we utilized balanced sets of Likert items at each measurement time to reduce the impact of the *acquiescence bias* (the tendency to agree with statements regardless of their content). At each measurement time, following the recoding of the two oppositely worded Likert items, the four Likert items were averaged to create a composite score defined on the interval scale [1,7] (1–3.75: pro-in-office-work opinion, 4: neutral opinion, 4.25–7: pro-remote-work opinion).

Drawing upon the characteristics of the composite score, we expertly determined the threshold value for opinion change as Δ = .5. In practical terms, this means that if the absolute difference between the composite scores at two measurement times is at least .5 (equivalent to a change in the evaluation of one Likert item by two points or of two Likert items by one point each in the same direction), we consider it a (notable) change in opinion. Conversely, if the difference is less than .5, there is no (notable) change in opinion.

### Procedure

The study was approved by the Ethics Committee of Management Center Innsbruck. Participants completed the study online, and the data were collected anonymously. The authors did not have access to information that could identify individual participants during or after data collection. In order not to reveal the real purpose of the study, it was presented to the participants as a *Survey of public opinion on remote work and work in a traditional office*. To make this more credible for the participants, we included several job-related questions in the filler tasks throughout the study. The median time spent on the study was 19.4 minutes (IQR = 9.8).

**Fig 1:**
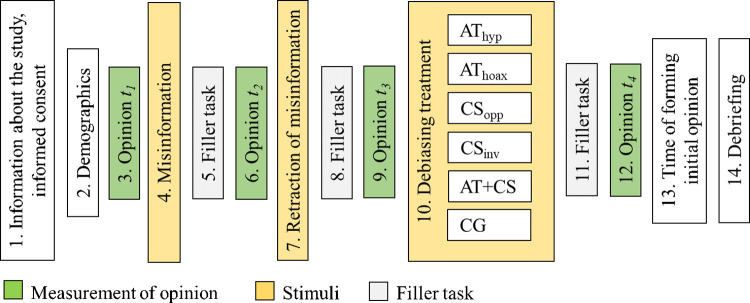
Study’s procedure.

The experimental procedure is illustrated in Fig 1. The experiment consisted of 14 steps:

1. Information about the study and informed consent: Participants were informed about the alleged purpose of the study and gave written informed consent for their participation by viewing a screen with informed consent information and clicking on the “agree” button.
2. Demographics: Participants provided their demographic information.
3. Measurement of initial opinion on the issue (measurement time *t*_*1*_)
4. Misinformation: Participants were exposed to (mis)information concerning remote and in-office work in the form of a blog article.
5. Filler task: Participants answered 21 questions concerning “calling in sick” behavior [39] (with slight modifications).
6. Measurement of opinion on the issue after the exposure to misinformation (measurement time *t*_*2*_).
7. Retraction of misinformation: Retraction was done in the spirit of the alleged purpose of the study.
8. Filler task: Participants completed a 16-item Equity Preference Questionnaire [40].
9. Measurement of opinion on the issue after the retraction (measurement time *t*_*3*_).
10. Debiasing treatment: Each participant was randomly assigned to one of the six debiasing treatment conditions (AT_hyp_, AT_hoax_, CS_opp_, CS_inv_, AT+CS, or CG) and exposed to the corresponding debiasing technique or the control treatment.
11. Filler task: Participants answered five questions concerning their job situation.
12. Measurement of opinion on the issue after the debiasing treatment (measurement time *t*_*4*_).
13. Question relevant for the subsequent study: Participants were asked to indicate the time (before or during the experiment) they formed their initial opinions on the issue.
14. Debriefing: Participants were debriefed about the real purpose of the study.

## Results

To test our hypotheses and research questions, we conducted a one-way ANOVA and t-tests comparing the mean opinions and the mean changes in opinions for different conditions and measurement times. Statistical analyses were performed using the Real Statistics Resource Pack for Excel.

### Exposure to misinformation and retraction

There was a neutral mean initial opinion on the issue at *t*_*1*_ (M_1_ = 4.08, SD = 1.19). After the exposure to (mis)information, there was a significant change in the mean opinion in the direction consistent with misinformation at *t*_*2*_ (*M*_2_ = 4.82, *SD* = 1.17), *t*_*2*,*1*_(875) = 19.76, *p* < .001, 95% confidence interval (CI) = [.67, .81], Cohen’s effect size d (d) = .67.

**Fig 2:**
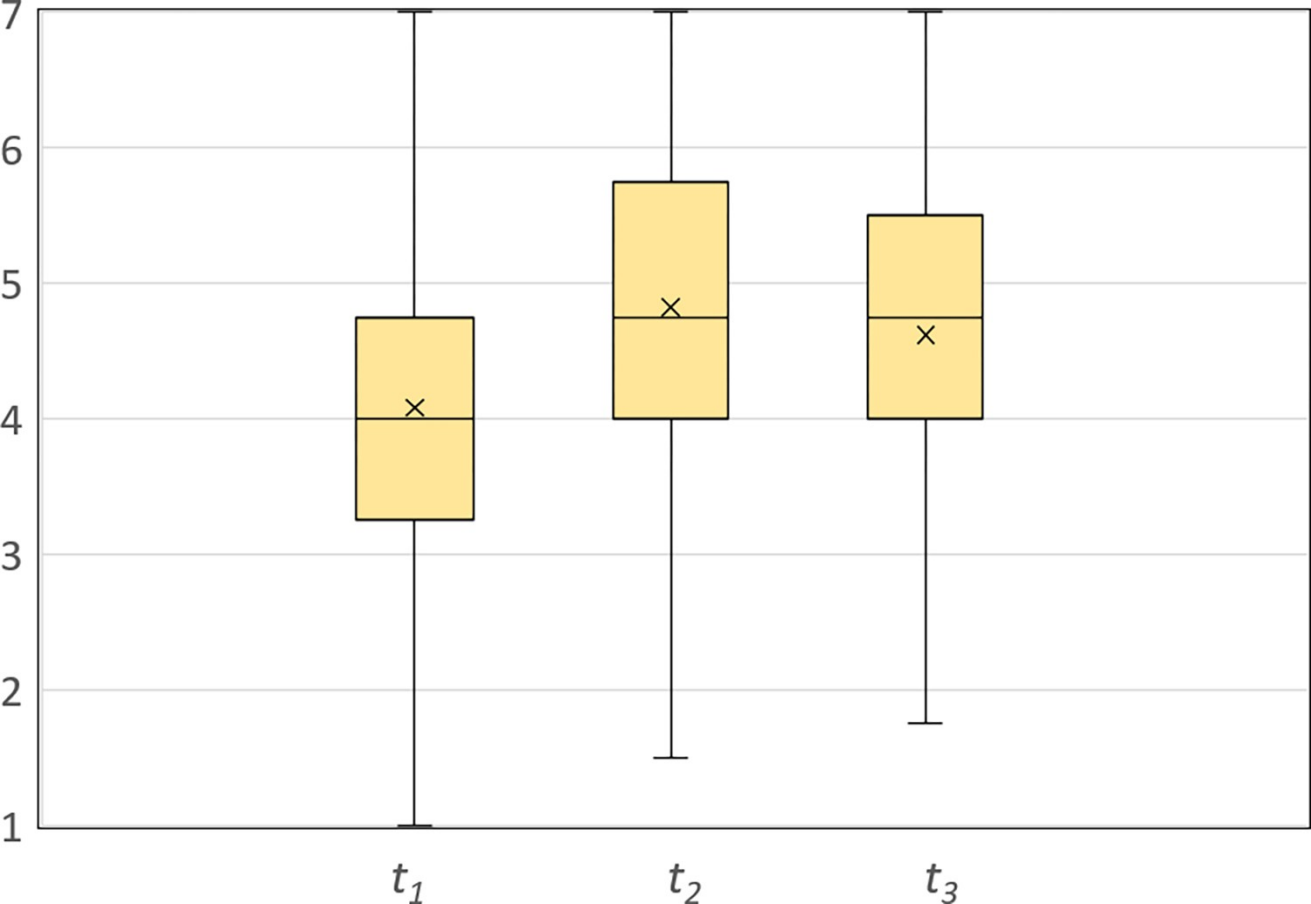
Boxplots of participants’ opinions (N = 876) at measurement times *t*_*1*_, *t*_*2*_, and *t*_*3*_. The top of the upper and the bottom of the lower whisker represent the maximum and minimum values, the top and bottom of the box represent the 75^th^ and 25^th^ percentiles, the line through the box represents the median, and the x marker represents the sample’s mean.

After the exposure to retraction, the mean opinion at *t*_*3*_ moved back in the direction of the mean initial opinion (*M*_3_ = 4.62, *SD* = 1.18), *t*_3,2_ (875) = -7.58, *p* < .001, CI = [-.26, -.15], d = .26, indicating that the retraction was effective in reducing the negative impact of misinformation. Despite the positive effect of retraction, the mean opinion at *t*_*3*_ was still significantly greater than the mean initial opinion at *t*_*1*_, *t*_*3*,*1*_(875) = 15.42, *p* < .001, CI = [.47, .60], d = .52, indicating the presence of BPB. The boxplots of participants’ opinions at the measurement times *t*_*1*_, *t*_*2*,_ and *t*_***3***_ are shown in Fig 2.

### Effectiveness of the debiasing techniques

To examine the effectiveness of debiasing techniques in mitigating BPB, we considered only the sample of participants experiencing BPB. In line with the definition of BPB and considering the expertly established threshold value Δ = .5 for opinion change, participants were considered to experience BPB when their opinions on the issue moved from the initial opinions at *t*_*1*_ in the direction congruent with misinformation at *t*_*2*_ (i.e., *o*_*t2*_ ≥ *o*_*t1*_ + .5) and their opinions remained biased even after the retraction of misinformation at *t*_*3*_ (i.e., *o*_*t3*_ ≥ *o*_*t1*_ + .5).

In the sample of 876 participants, 504 participants (58%) showed biased opinions on the issue after encountering misinformation (i.e., *o*_*t2*_ ≥ *o*_*t1*_ + .5), and 364 (42%) experienced BPB after the retraction of misinformation (*o*_*t3*_ ≥ *o*_*t1*_ + .5). The sample of N = 364 participants who experienced BPB comprised 184 females and 180 males. The mean age of participants experiencing BPB was 27.0 years (SD = 5.3 years). In terms of education, 178 participants completed high school, 184 had a university education, and two did not finish high school. Regarding occupation, 242 participants were employed, 57 were unemployed, and 65 were students.

For assessing the effectiveness of the debiasing techniques (AT_hyp_, AT_hoax_, CS_opp_, CS_inv_, and AT+CS) in mitigating BPB, we used the difference between the opinions after the exposure to a debiasing treatment at the measurement time *t*_*4*_ and the initial opinions at the measurement time *t*_*1*_ in the sample of N = 364 participants experiencing BPB and compared the corresponding debiasing treatment group with the control group (CG). Table 1 shows the relevant statistics for the debiasing treatment conditions at the measurement times *t*_*1*_, *t*_*2*_, *t*_*3*,_ and *t*_*4*_ and the differences in opinions between the measurement times *t*_*4*_ and *t*_*1*_.

**Table 1:** Opinion means and standard deviations at measurement times *t*_*1*_, *t*_*2*_, *t*_*3*,_ and *t*_*4*_ and t-tests on the differences in opinions between measurement times *t*_*4*_ and *t*_*1*_ for debiasing treatment groups in the sample with BPB (N = 364).

Group	N	Means				Standard deviations				Differences in opinions between *t*_*4*_ and *t*_*1*_					
		M_1_	M_2_	M_3_	M_4_	SD_1_	SD_2_	SD_3_	SD_4_	M_4-1_	CI_95%_	SD	t-stat	p-value	Cohen’s d
CG	46	3.65	5.26	5.08	5.07	.88	.84	.91	.89	1.41	[1.15,1.67]	.88	10.92	< .001	1.61
AT_hyp_	74	3.58	5.25	5.06	4.44	1.02	1.00	1.08	1.03	.85	[.68,1.03]	.76	9.64	< .001	1.12
AT_hoax_	52	3.41	4.99	4.68	4.25	1.03	1.22	1.08	1.13	.83	[.60,1.07]	.84	7.15	< .001	.99
CS_opp_	56	3.50	5.11	4.99	4.05	1.09	1.05	1.06	1.12	.55	[.33,.77]	.84	4.90	< .001	.65
CS_inv_	63	3.57	5.35	5.04	4.01	.91	1.00	.98	1.23	.44	[.18,.72]	1.04	3.41	.001	.43
AT+CS	73	3.69	5.19	4.98	3.80	1.12	1.03	1.04	1.11	.11	[-.13,.35]	1.02	.92	.36	.11
AT (AT_hyp_ or AT_hoax)_	126	3.51	5.14	4.91	4.36	1.02	1.10	1.10	1.07	.84	[.70,.98]	.79	11.99	< .001	1.07
CS (CS_opp_ or CS_inv)_	119	3.54	5.23	5.02	4.03	1.00	1.03	1.01	1.18	.49	[.32,.67]	.95	5.70	< .001	.52

The AT group is obtained by merging the AT_hyp_ and AT_hoax_ treatment groups.

The CS group is obtained by merging the CS_inv_ and CS_opp_ treatment groups.

To explore RQ1 and RQ2, we conducted two-sample t-tests on the differences in opinions between *t*_*4*_ and *t*_*1*_ for the two variants of AT and CS. There was no significant difference in the extent of BPB between AT_hyp_ and AT_hoax_, *t*(124) = .14, *p* = .89, CI = [-.26, .30], d = .02 (RQ1). Analogously, there was also no significant difference in the extent of BPB between CS_inv_ and CS_opp_, *t*(117) = .60, *p* = .55, CI = [-.45, .24], d = .11 (RQ2). In the follow-up analyses, we, therefore, did not further distinguish between the specific variants of the AT and CS techniques. Instead, we combined the AT_hyp_ and AT_hoax_ treatment groups into the AT group and the CS_inv_ and CS_opp_ treatment groups into the CS group. The relevant statistics for the resulting AT and CS treatment groups are provided in the last two rows of Table 1.

**Fig 3:**
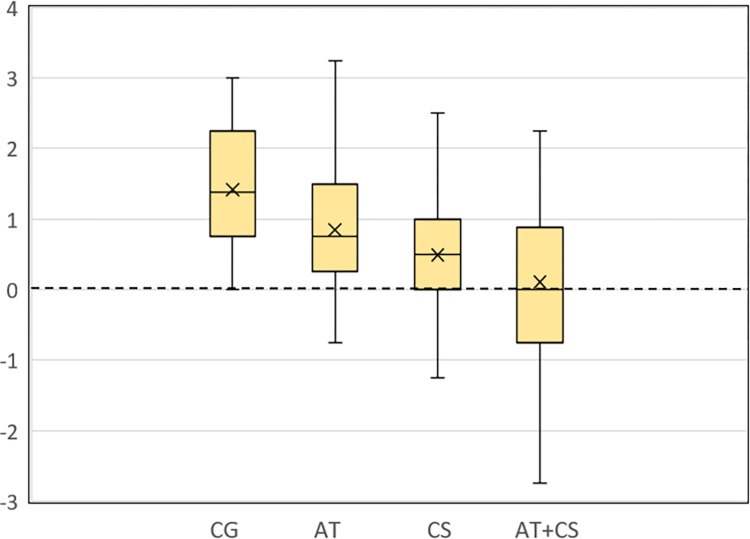
Boxplots of differences in opinions between measurement times *t*_*4*_ and *t*_*1*_ for debiasing treatment groups. The top of the upper and the bottom of the lower whisker represent the maximum and minimum values, the top and bottom of the box represent the 75^th^ and 25^th^ percentiles, the line through the box represents the median, and the x marker represents the sample’s mean. The dashed horizontal line at 0 represents the ideal scenario where the opinion after the debiasing treatment at *t*_*4*_ is the same as the initial opinion at *t*_*1*_, i.e., there is no BPB.

To test H1, H2, H3, and H4 and explore RQ3, we conducted a one-way ANOVA on the differences in opinions between the measurement times *t*_*4*_ and *t*_*1*_ and the Tukey-Kramer post hoc test. There was a significant effect of the debiasing treatments, *F*(3,360) = 22.82, *p* < .001, η^2^_p_ = .16. Tukey-Kramer post hoc test (Table 2) showed that all three debiasing techniques AT, CS, and AT+CS, were effective in mitigating BPB compared to the CG and that CS was more effective than AT, which supports hypotheses H1, H2, H3, and H4. As for research question RQ3, the Tukey-Kramer post hoc test showed that AT+CS was more effective than the single techniques AT and CS. Fig 3 shows the boxplots of the differences in opinions between the measurement times *t*_*4*_ and *t*_*1*_ for the debiasing treatment groups.

**Table 2:** Tukey-Kramer post-hoc test on the differences in opinions between measurement times *t*_*4*_ and *t*_*1*_ for debiasing treatment groups in the sample with BPB (N = 364).

Group 1	Group 2	M	CI_95%_	q-stat	p-value	Cohen’s d
AT	CG	.57	[.17,.97]	5.19	.002	.63
CS	CG	.92	[.52,1.32]	8.30	< .001	1.02
AT+CS	CG	1.30	[.87,1.74]	10.86	< .001	1.45
CS	AT	.35	[.05,.65]	4.29	.014	.39
AT+CS	CS	.38	[.04,.73]	4.05	.023	.43
AT+CS	AT	.73	[.39,1.08]	7.82	< .001	.81

## Discussion

### Belief perseverance bias

Consistent with prior research, we observed an impact of misinformation on opinions and the presence of BPB after the retraction of misinformation [4,33,36,41]. In our study, 58% of the participants were influenced by misinformation, and 42% experienced BPB after the retraction of misinformation. These percentages are notably lower than in the study by Siebert and Siebert [4] (85% and 68.5%, respectively). A possible explanation for these differences is the difference between the issues subject to misinformation. Siebert and Siebert used a constructed issue concerning a fictitious social theory on which most, if not all, participants were likely to have no preexisting opinions [2,4]. Our study used a real-life issue for which most participants reported preexisting opinions. The differences between the studies are consistent with the fact that individuals with preexisting opinions on a specific issue tend to be more resistant to change than individuals without preexisting opinions [42].

### Effectiveness of the debiasing techniques

We studied the effectiveness of the AT and CS debiasing techniques, as proposed by Siebert and Siebert [4], along with their modifications and a combination in mitigating BPB after the retraction of misinformation related to a real-life issue. Consistent with the findings of Siebert and Siebert [4], AT and CS demonstrated effectiveness in mitigating BPB (H1 and H2). These results contribute further evidence for the effectiveness of the AT and CS debiasing techniques in mitigating BPB and highlight their potential applicability to real-life misinformation. Future research should further explore the effectiveness of these debiasing techniques across a broader spectrum of real-life issues prone to misinformation.

In line with Siebert and Siebert’s study [4], our findings indicated that CS was more effective in mitigating BPB than AT (H3). This outcome aligns with previous research indicating that refutations tend to be more effective than forewarnings [1], although it has to be noted that our AT—a specific warning against BPB—was applied as a debunking intervention. Our result thus supports Siebert and Siebert’s recommendation to prioritize CS as the primary choice for achieving optimal debiasing effects, while AT becomes a suitable alternative when aiming to reduce debiasing providers’ efforts or when no suitable counter-arguments are available for CS. However, despite CS’s greater effectiveness, it did not completely eliminate BPB, which is consistent with prior research indicating that corrections may not entirely eliminate the impact of misinformation [2].

Our study found no discernible differences in the effectiveness between the original variants of AT and CS proposed by Siebert and Siebert [4] and the new variants proposed in this paper (RQ1 and RQ2). However, as our examination focused solely on one specific real-life issue subject to misinformation, it remains unclear to what extent this finding can be generalized to other real-life misinformation scenarios. Therefore, future research should aim to compare the effectiveness of the AT and CS variants on various real-life issues subject to misinformation.

As both variants of AT and CS are effective in mitigating BPB, in practice, providers of debiasing may choose a specific variant for addressing particular misinformation, taking into account the unique features of each case. For instance, the CS_opp_ or CS_inv_ variants might be selected based on the kind of available evidence refuting the specific claim of misinformation. The AT_hoax_ variant, which uses the alleged relationship between the measles-mumps-rubella vaccination and autism to illustrate the negative impact of BPB, could be well-suited for debunking misinformation related to medical and health issues. Future research could explore the effectiveness of additional AT variants, illustrating the negative impact of BPB on various real-life examples (such as from science, politics, or health).

Our study contributes significantly to the limited research on combining techniques to reduce the negative impact of misinformation. We discovered that the combination of AT and CS is not only effective in mitigating BPB (H4) but also more effective than the single debiasing techniques (RQ3). Our findings align with Hameleers’ research, which found that combining a news media literacy intervention with a detailed refutation is more effective in reducing the issue agreement and the perceived accuracy of misinformation than single interventions [8]. However, our results differ from Vraga et al.’s findings, which indicated that combining news literacy tweets with short-format refutations is not more effective than short-format refutations alone [30]. These discrepancies could be attributed to differences in the types and formats of corrective interventions [8]. Notably, our AT and CS interventions, along with Hameleers’ news media literacy and refutation interventions [8], are all long-format (each about a half A4 page long), while Vraga et al.’s news literacy tweets and refutations [30] are short-format (each consisting of just one sentence). This suggests that short-format interventions, which can only provide a brief awareness or news literacy alert and a brief refutation without going into details, might be less persuasive than long-format interventions, which offer more detailed awareness or news literacy training and detailed refutations supported with arguments. Therefore, future research should explore the impact of intervention length and detail on the effectiveness of their combinations in reducing the negative impact of misinformation. Additionally, validation of our findings on other real-life issues subject to misinformation would be valuable.

We employed the AT technique as a debunking intervention, as Siebert and Siebert [4] did. However, due to the universal formulation of AT, which does not require adaptions to specific misinformation, the AT technique can also be applied as a prebunking intervention [4]. Previous research has demonstrated that warnings, inoculations, and media and news literacy interventions, when applied as prebunking interventions, can effectively reduce the negative impact of misinformation [7,8,12,13,16]. Nevertheless, Tay et al. found that inoculation is less effective in reducing the negative impact of misinformation when employed as a prebunking intervention compared to a debunking intervention [25]. Additionally, some studies have suggested that prebunking interventions may lead to less accurate identification and decreased belief in accurate information [43,44]. Hence, future research should examine the effectiveness of AT in mitigating BPB when applied as a prebunking intervention and explore potential side effects on beliefs in accurate information.

### Limitations

Our study has several limitations that warrant attention in future research. Firstly, our sample was younger and more educated compared to the general population. Since different demographic groups may respond differently to misinformation, its retraction, and debiasing techniques, validating the findings on a nationally representative sample is essential. Secondly, despite the inclusion of filler tasks between stimuli (misinformation, retraction, and debiasing treatments) and opinion measurements, the retraction of misinformation and the debiasing occurred shortly after exposure to misinformation, following the common practice in studies on reducing the negative impact of misinformation and mitigating BPB. This design limitation restricted us to capturing only short-term effects. Consequently, the generalizability of our findings to real-world scenarios, where the time lapse between the exposure to (mis)information and misinformation retraction is typically longer, remains uncertain. Future research should, therefore, investigate the long-term effects of misinformation retraction and debiasing techniques on BPB. Lastly, as with any social science experiment, potential experimenter demand effects cannot be entirely eliminated. Studies measuring the impact of misinformation and its correction by soliciting participants’ opinions, agreement with misinformation, or perceived accuracy or reliability of statements may prompt participants to deduce the study’s true purpose and shape their responses to align with researchers’ hypotheses. Nevertheless, existing research suggests that online survey experiments are robust to experimenter demand effects [45].

## Supporting information

S1 Appendix Supplemental material. (DOCX)

## References

[1] Walter N, Murphy ST. How to unring the bell: A meta-analytic approach to correction of misinformation. Communication Monographs. 2018;85:423–41. doi: 10.1080/03637751.2018.1467564

[2] Walter N, Tukachinsky R. A Meta-Analytic Examination of the Continued Influence of Misinformation in the Face of Correction: How Powerful Is It, Why Does It Happen, and How to Stop It? Communication Research. 2020;47:155–77. doi: 10.1177/0093650219854600

[3] Chan M-PS, Jones CR, Hall Jamieson K, Albarracín D. Debunking: A Meta-Analysis of the Psychological Efficacy of Messages Countering Misinformation. Psychological Science. 2017;28:1531–46. doi: 10.1177/0956797617714579 28895452 PMC5673564

[4] Siebert J, Siebert UJ. Effective mitigation of the belief perseverance bias after the retraction of misinformation: Awareness training and counter-speech. PLOS ONE 2023. doi: 10.1371/journal.pone.0282202 36888583 PMC9994702

[5] Lewandowsky S, van der Linden S. Countering Misinformation and Fake News Through Inoculation and Prebunking. European Review of Social Psychology. 2021;32:348–84. doi: 10.1080/10463283.2021.1876983

[6] Lewandowsky S, Ecker UKH, Seifert CM, Schwarz N, Cook J. Misinformation and Its Correction: Continued Influence and Successful Debiasing. Psychol Sci Public Interest. 2012;13:106–31. doi: 10.1177/1529100612451018 26173286

[7] Clayton K, Blair S, Busam JA, Forstner S, Glance J, Green G, et al. Real Solutions for Fake News? Measuring the Effectiveness of General Warnings and Fact-Check Tags in Reducing Belief in False Stories on Social Media. Polit Behav. 2020;42:1073–95. doi: 10.1007/s11109-019-09533-0

[8] Hameleers M. Separating truth from lies: comparing the effects of news media literacy interventions and fact-checkers in response to political misinformation in the US and Netherlands. Information, Communication & Society. 2022;25:110–26. doi: 10.1080/1369118X.2020.1764603

[9] Lewandowsky S, Yesilada M. Inoculating against the spread of Islamophobic and radical-Islamist disinformation. Cogn. Research. 2021;6:57. doi: 10.1186/s41235-021-00323-z 34410513 PMC8374109

[10] Roozenbeek J, van der Linden S. Fake news game confers psychological resistance against online misinformation. Palgrave Commun. 2019;5:1–10. doi: 10.1057/s41599-019-0279-9

[11] Roozenbeek J, van der Linden S. The fake news game: actively inoculating against the risk of misinformation. Journal of Risk Research. 2019;22:570–80. doi: 10.1080/13669877.2018.1443491

[12] Pennycook G, Bear A, Collins ET, Rand DG. The Implied Truth Effect: Attaching Warnings to a Subset of Fake News Headlines Increases Perceived Accuracy of Headlines Without Warnings. Management Science. 2020;66:4944–57. doi: 10.1287/mnsc.2019.3478

[13] Tully M, Vraga EK, Bode L. Designing and Testing News Literacy Messages for Social Media. Mass Communication and Society. 2020;23:22–46. doi: 10.1080/15205436.2019.1604970

[14] Ecker UKH, Lewandowsky S, Fenton O, Martin K. Do people keep believing because they want to? Preexisting attitudes and the continued influence of misinformation. Mem Cogn. 2014;42:292–304. doi: 10.3758/s13421-013-0358-x 24005789

[15] Ecker UKH, Lewandowsky S, Swire B, Chang D. Correcting false information in memory: manipulating the strength of misinformation encoding and its retraction. Psychon Bull Rev. 2011;18:570–8. doi: 10.3758/s13423-011-0065-1 21359617

[16] Ecker UKH, Lewandowsky S, Tang DTW. Explicit warnings reduce but do not eliminate the continued influence of misinformation. Mem Cogn. 2010;38:1087–100. doi: 10.3758/MC.38.8.1087 21156872

[17] Rich PR, Zaragoza MS. The continued influence of implied and explicitly stated misinformation in news reports. Journal of Experimental Psychology: Learning, Memory, and Cognition. 2016;42:62–74. doi: 10.1037/xlm0000155 26147670

[18] Anderson CA. Inoculation and counterexplanation: Debiasing techniques in the perseverance of social theories. Social Cognition. 1982;1:126–39. doi: 10.1521/soco.1982.1.2.126

[19] Cobb MD, Nyhan B, Reifler J. Beliefs Don’t Always Persevere: How Political Figures Are Punished When Positive Information about Them Is Discredited. Political Psychology. 2013;34:307–26. doi: 10.1111/j.1467-9221.2012.00935.x

[20] Green MC, Donahue JK. Persistence of Belief Change in the Face of Deception: The Effect of Factual Stories Revealed to Be False. Media Psychology. 2011;14:312–31. doi: 10.1080/15213269.2011.598050

[21] Anderson CA, Sechler ES. Effects of explanation and counterexplanation on the development and use of social theories. Journal of Personality and Social Psychology. 1986;50:24–34. doi: 10.1037/0022-3514.50.1.24

[22] Johnson HM, Seifert CM. Sources of the continued influence effect: When misinformation in memory affects later inferences. Journal of Experimental Psychology: Learning, Memory, and Cognition. 1994;20:1420–36. doi: 10.1037/0278-7393.20.6.1420

[23] Nyhan B, Reifler J. Displacing Misinformation about Events: An Experimental Test of Causal Corrections. J Exp Polit Sci. 2015;2:81–93. doi: 10.1017/xps.2014.22

[24] Swire B, Ecker UKH, Lewandowsky S. The role of familiarity in correcting inaccurate information. Journal of Experimental Psychology: Learning, Memory, and Cognition. 2017;43:1948–61. doi: 10.1037/xlm0000422 28504531

[25] Tay LQ, Hurlstone MJ, Kurz T, Ecker UKH. A comparison of prebunking and debunking interventions for implied versus explicit misinformation. British Journal of Psychology. 2022;113:591–607. doi: 10.1111/bjop.12551 34967004

[26] Ecker UKH, Lewandowsky S, Chadwick M. Can corrections spread misinformation to new audiences? Testing for the elusive familiarity backfire effect. Cogn. Research. 2020;5:41. doi: 10.1186/s41235-020-00241-6 32844338 PMC7447737

[27] Anderson CA. Belief Perseverance. In: Baumeister RF, Vohs KD, editors. Encyclopedia of social psychology. Los Angeles, London: Sage; 2007.

[28] Anderson CA, Lepper MR, Ross L. Perseverance of social theories: The role of explanation in the persistence of discredited information. Journal of Personality and Social Psychology. 1980;39:1037–49. doi: 10.1037/h0077720

[29] McGuire WJ. The Effectiveness of Supportive and Refutational Defenses in Immunizing and Restoring Beliefs Against Persuasion. Sociometry. 1961;24:184. doi: 10.2307/2786067

[30] Vraga EK, Bode L, Tully M. Creating News Literacy Messages to Enhance Expert Corrections of Misinformation on Twitter. Communication Research. 2022;49:245–67. doi: 10.1177/0093650219898094

[31] Hameleers M, van der Meer TGLA. Misinformation and Polarization in a High-Choice Media Environment: How Effective Are Political Fact-Checkers? Communication Research. 2020;47:227–50. doi: 10.1177/0093650218819671

[32] Ecker UKH, Sze BKN, Andreotta M. Corrections of political misinformation: no evidence for an effect of partisan worldview in a US convenience sample. Philos Trans R Soc Lond B Biol Sci. 2021;376:20200145. doi: 10.1098/rstb.2020.0145 33612006 PMC7934973

[33] Ecker UKH, Ang LC. Political Attitudes and the Processing of Misinformation Corrections. Political Psychology. 2019;40:241–60. doi: 10.1111/pops.12494

[34] Swire B, Berinsky AJ, Lewandowsky S, Ecker UKH. Processing political misinformation: comprehending the Trump phenomenon. R Soc Open Sci. 2017;4:160802. doi: 10.1098/rsos.160802 28405366 PMC5383823

[35] Huang H. A War of (Mis)Information: The Political Effects of Rumors and Rumor Rebuttals in an Authoritarian Country. Brit. J. Polit. Sci. 2017;47:283–311. doi: 10.1017/s0007123415000253

[36] Maegherman E, Ask K, Horselenberg R, van Koppen PJ. Law and order effects: on cognitive dissonance and belief perseverance. Psychiatry, Psychology and Law. 2022;29:33–52. doi: 10.1080/13218719.2020.1855268 35693388 PMC9186347

[37] Bode L, Vraga EK. In Related News, That Was Wrong: The Correction of Misinformation Through Related Stories Functionality in Social Media. J Commun. 2015;65:619–38. doi: 10.1111/jcom.12166

[38] Vraga EK, Bode L. Using Expert Sources to Correct Health Misinformation in Social Media. Science Communication. 2017;39:621–45. doi: 10.1177/1075547017731776

[39] Hauge KE, Ulvestad ME. Having a bad attitude? The relationship between attitudes and sickness absence. IZA J Labor Policy. 2017;6:1–27. doi: 10.1186/s40173-017-0088-y

[40] Sauley KS, Bedeian AG. Equity Sensitivity: Construction of a Measure and Examination of Its Psychometric Properties. Journal of Management. 2000;26:885–910. doi: 10.1177/014920630002600507

[41] Zhou Y, Shen L. Confirmation Bias and the Persistence of Misinformation on Climate Change. Communication Research. 2022;49:500–23. doi: 10.1177/00936502211028049

[42] Howe LC, Krosnick JA. Attitude Strength. Annu. Rev. Psychol. 2017;68:327–51. doi: 10.1146/annurev-psych-122414-033600 27618943

[43] van Duyn E, Collier J. Priming and Fake News: The Effects of Elite Discourse on Evaluations of News Media. Mass Communication and Society. 2019;22:29–48. doi: 10.1080/15205436.2018.1511807

[44] Buczel M, Szyszka PD, Siwiak A, Szpitalak M, Polczyk R. Vaccination against misinformation: The inoculation technique reduces the continued influence effect. PLOS ONE. 2022;17:e0267463. doi: 10.1371/journal.pone.0267463 35482715 PMC9049321

[45] Mummolo J, Peterson E. Demand Effects in Survey Experiments: An Empirical Assessment. Am Polit Sci Rev. 2019;113:517–29. doi: 10.1017/s0003055418000837

