# Supplementary material for: Enhancing misinformation correction: New variants and a combination of awareness training and counter-speech to mitigate belief perseverance bias
Source: PLoS One. 2024 Feb 16;19(2):e0299139. doi: 10.1371/journal.pone.0299139 (PMC10871482; doi:10.1371/journal.pone.0299139)
Supplement: S1 Appendix — (DOCX) [file pone.0299139.s001.docx]

# Online appendix

**Enhancing misinformation correction:**

**New variants and a combination of awareness training and counter-speech to mitigate belief perseverance bias**

Jana Siebert^1^, Johannes Ulrich Siebert^2^*

^1^ Department of Economic and Managerial Studies, Faculty of Arts, Palacky University Olomouc, 779 00 Olomouc, Czech Republic

^2^ Department of Business and Management, Management Center Innsbruck, 6020 Innsbruck, Austria

* Corresponding author

E-mail: [johannes.siebert@mci.edu](mailto:johannes.siebert@mci.edu)

**[Appendix A: Preparatory study](#appendixA)**

[**Appendix B: Measures of opinion**](#appendixB)

[**Appendix C: Misinformation and retraction stimuli**](#appendixC)

[**Appendix D: Debiasing treatment conditions**](#appendixD)

# Appendix A: Preparatory study

The preparatory study aims to: 1) assess the suitability of two misinformation stimuli for biasing participants’ opinions on the issue concerning the relationship between the work location (remove vs. in-office) and companies’ productivity and efficiency (short *opinion on the issue*) and inducing belief perseverance bias (BPB) after the retraction of misinformation and 2) develop and validate measures of opinion on the issue.

## Method

### Study design

We used a web-based pretest-posttest between-subject experimental design. The participants were randomly allocated to one of two misinformation conditions (pro-in-office-work misinformation vs. pro-remote-work misinformation).

### Participants

Participants were recruited by online survey provider Qualtrics^©^ in the UK. We decided to focus our study on young adults. Thus, participants had to be between 18 and 35 years old to be included in the study. Furthermore, only participants with good English were eligible for the study.

We collected data from 105 participants. The sample consisted of 51 females and 54 males. The mean age for participants was 28 years (SD = 5.1 years). In terms of education, 59 participants attained a university education, 45 attained a high school education, and one did not finish high school. Regarding employment, 75 participants were employed, 17 were unemployed, and 13 were students.

### Procedure

Participants completed the study online, and the data were collected anonymously. The median time spent on the study was 18.3 minutes (IQR = 7.5). In order not to reveal the real purpose of the study, it was presented to the participants as a *Survey of public opinion on remote work and work in a traditional office*. To make this more credible for the participants, we included several job-related questions in the filler tasks throughout the study. At the beginning, participants were informed about the alleged purpose of the study, gave informed consent for their participation, and provided demographic information. Next, participants provided their initial opinions on the issue (measurement time *t_1_*). Afterward, participants were randomly assigned to one of two misinformation conditions (pro-in-office-work misinformation vs. pro-remote-work misinformation) and exposed to the corresponding stimulus. This was followed by a filler task. In the next, participants completed 19 Likert items (always one from each pair of oppositely worded Likert items) to be validated in the study. The order of the Likert items was randomized for each participant to reduce the question order bias. Afterward, participants again provided their opinions on the issue (measurement time *t_2_*). This was followed by the retraction of misinformation, which was done in the spirit of the alleged purpose of the study, and another filler task, in which participants answered five questions concerning their job situation. Afterward, participants provided their opinions on the issue once more (measurement time *t_3_*). In the end, they were debriefed about the real purpose of the study.

### Stimuli

Participants in the pro-remote-work misinformation condition were exposed to misinformation suggesting that companies with employees working remotely are more productive and effective than companies with employees working in an office, while participants in the pro-in-office-work misinformation condition were exposed to misinformation suggesting the opposite (see Supplemental Appendix C for misinformation stimuli). The (mis)information took the form of a one-page blog article that was entirely fabricated. The article summarized the results of an international study comparing remote and in-office work in terms of benefits for companies.

### Dependent variable – opinion on the issue

We created two oppositely worded incomplete direct-comparison statements about the companies’ productivity and efficiency (“In my opinion, companies with employees working remotely tend to be _____ companies with employees working in an office.” and “In my opinion, companies with employees working in an office tend to be _____ companies with employees working remotely.”(oppositely worded)) that were to be completed by choosing from the list of 9 items (1 – extremely less productive and efficient than, 5 – as productive and efficient as, 9 – extremely more productive and efficient than).

We measured participants’ opinions on the issue using the direct-comparison measure three times during the experiment to detect BPB (measurement time *t_1_*: initial opinion, *t_2_*: opinion after the exposure to misinformation, *t_3_*: opinion after the retraction). Each participant was assigned one randomly selected direct-comparison statement at each measurement time.

The direct-comparison measure could be sufficient if we intended to measure participants’ opinions only once within the main study. However, following the approach used in Siebert and Siebert (2023)’s study, we measure participants’ opinions several times and plan to use different sets of measures at each measurement time. To achieve this, we necessitate a sufficient number of suitable measurement items. Thus, in this study, we use the direct-comparison measure as a reference measure for validating other measures of participants’ opinions on the issue.

### Validation of Likert items for measuring opinion

We created a list of 19 pairs of oppositely worded Likert items related to the work location (see Supplemental Appendix B) to be assessed on a 7-point scale (1 – completely disagree, 4 – neither agree nor disagree, 7 – completely agree). One item in each pair had a pro-remote-work formulation, while the other item (oppositely worded) had a pro-in-office-work formation. Each participant was administered a set of 19 Likert items (always one from each pair of oppositely worded Likert items) at the measurement time *t_2_* in the study together with one randomly chosen direct-comparison measure. The order of the measurement items was randomized for each participant to reduce the question order bias.

## Results and discussion

We used a series of t-tests comparing the mean opinions at different measurement times for the two misinformation conditions to examine the impact of misinformation and its retraction on changes in opinions and detect BPB. Further, we performed correlation analysis to assess the concurrent validity of the Likert items.

### Validation of the misinformation stimuli

The mean initial opinion on the issue was slightly pro-remote-work (direct-comparison measure on the 9-point ordinal scale at *t_1_*: M_1_= 4.74, SD = 1.94). After the exposure to misinformation, there was a significant change in the mean opinion at *t_2_* in the direction consistent with misinformation both in the pro-remote-work condition (M_2_= 6.06, SD = 1.70), *t_1,2_*(52) = -3.88, *p* = 1.5E-4, *d* = 0.53, and the pro-in-office-work condition (M_2_= 3.44, SD = 1.82), *t_1,2_*(51) = 12.66, *p* = 4.8E-4, *d* = 0.49.

After the exposure to retraction, the mean opinion at *t_3_* moved back in the direction of the mean initial opinion both in the pro-remote-work condition (M_3_= 5.74, SD = 1.79) and the pro-in-office-work condition (M_3_= 3.56, SD = 1.63). Nevertheless, the mean opinion at *t_3_* was still significantly different from the mean initial opinion in both conditions (pro-remote-work: *t_1,3_*(52) = - 4.18, *p* = 5.6E-5, *d* = 0.57, pro-in-office-work: *t_1,3_*(51) = 3.71, *p* = 2.6E-4, *d* = 0.51), which indicates the presence of BPB. These results show that both the pro-remote-work misinformation and the pro-in-office-work misinformation impact people’s opinions on the issue, and this impact persists even after the retraction of misinformation.

### Validation of the measures

Correlations with the direct-comparison measure at the measurement time *t_2_* were analyzed to assess the concurrent validity of the Likert items. Since the scales for the direct-comparison measure and the Likert items are ordinal, Spearman’s coefficient *ρ* was applied.

First, we recoded the 19 Likert items with the pro-in-office-work formulation and merged the oppositely formulated Likert items in each pair. That is, we were further working with 19 Likert items (not pairs). The correlation analysis showed moderate to strong correlations of the Likert items with the direct-comparison measure. In particular, except for two Likert items, LIK_18_ and LIK_19_ (0.43 < *ρ* < 0.47, *p* < 3.5E-6), the correlations of all other Likert items with the direct-comparison measure were above 0.50, ranging from 0.53 to 0.70 (M = 0.64, SD = 0.04, *p* < 9.8E-10). By removing the Likert items LIK_18_ and LIK_19_ from the set, the correlations among the remaining 17 Likert items ranged from 0.48 to 0.90 (M = 0.73, SD = 0.08). By additionally removing the Likert item LIK_17_, the correlations among the remaining 16 Likert items were strong, ranging from 0.59 to 0.90 (M = 0.75, SD = 0.07). The correlation analysis showed concurrent validity of 16 pairs of Likert items. These were, therefore, adopted as valid measures of participants’ opinions on the issue to be used in the main study.

# Appendix B: Measures of opinion

Table 1: 19 pairs of oppositely worded Likert items tested in the preparatory study. Subscripts R and O indicate the pro-remote-work and pro-in-office-work formulation, respectively. All Likert items, except for the pairs LIK_17_, LIK_18_, and LIK_19,_ were adopted as valid measures of opinion on the issue and used in the main study.

| 1. LIK_1R_ | Employees working remotely tend to be more productive than employees working in an office. |
| --- | --- |
| 1. LIK_1O_ | Employees working in an office tend to be more productive than employees working remotely. |
| 1. LIK_2R_ | Employees working remotely tend to have a better performance than employees working in an office. |
| 1. LIK_2O_ | Employees working remotely tend to have a better performance than employees working in an office. |
| 1. LIK_3R_ | Employees working remotely tend to meet employer’s expectations better than employees working in an office. |
| 1. LIK_3O_ | Employees working in an office tend to meet employer’s expectations better than employees working remotely. |
| 1. LIK_4R_ | Employees working remotely tend to be more engaged at work than employees working in an office. |
| 1. LIK_4O_ | Employees working in an office tend to be more engaged at work than employees working remotely. |
| 1. LIK_5R_ | Employees working remotely tend to be more committed to their company than employees working in an office. |
| 1. LIK_5O_ | Employees working in an office tend to be more committed to their company than employees working remotely. |
| 1. LIK_6R_ | Employees working remotely tend to get more work done than employees working in an office. |
| 1. LIK_6O_ | Employees working in an office tend to get more work done than employees working remotely. |
| 1. LIK_7R_ | Employees working remotely tend to be more motivated at work than employees working in an office. |
| 1. LIK_7O_ | Employees working in an office tend to be more motivated at work than employees working remotely. |
| 1. LIK_8R_ | Companies with employees working remotely tend to be more prosperous than companies with employees working in an office. |
| 1. LIK_8O_ | Companies with employees working in an office tend to be more prosperous than companies with employees working remotely. |
| 1. LIK_9R_ | Companies with employees working remotely tend to be more efficient than companies with employees working in an office. |
| 1. LIK_9O_ | Companies with employees working in an office tend to be more efficient than companies with employees working remotely. |
| 1. LIK_10R_ | Companies with employees working remotely tend to perform better than companies with employees working in an office. |
| 1. LIK_10O_ | Companies with employees working in an office tend to perform better than companies with employees working remotely. |
| 1. LIK_11R_ | Companies with employees working remotely tend to be more successful than companies with employees working in an office. |
| 1. LIK_11O_ | Companies with employees working in an office tend to be more successful than companies with employees working remotely. |
| 1. LIK_12R_ | Companies with employees working remotely tend to be more productive than companies with employees working in a traditional office. |
| 1. LIK_12O_ | Companies with employees working in an office tend to be more productive than companies with employees working remotely. |
| 1. LIK_13R_ | Remote work is for companies more beneficial than work in an office. |
| 1. LIK_13O_ | Work in an office is for companies more beneficial than remote work. |
| 1. LIK_14R_ | Companies with employees working remotely tend to achieve their goals more easily than companies with employees working in a traditional office. |
| 1. LIK_14O_ | Companies with employees working in an office tend to achieve their goals more easily than companies with employees working remotely. |
| 1. LIK_15R_ | If I were a company owner, I would prefer to let the employees work remotely rather than in an office. |
| 1. LIK_15O_ | If I were a company owner, I would prefer to let the employees work in an office rather than remotely. |
| 1. LIK_16R_ | Costs caused by low-productivity employees tend to be lower for companies with employees working remotely than for companies with employees working in an office. |
| 1. LIK_16O_ | Costs caused by low-productivity employees tend to be lower for companies with employees working in an office than for companies with employees working remotely. |
| 1. LIK_17R_ | Companies with employees working remotely tend to have a lower employee turnover rate than companies with employees working in an office. |
| 1. LIK_17O_ | Companies with employees working in an office tend to have a lower employee turnover rate than companies with employees working remotely. |
| 1. LIK_18R_ | Companies with employees working remotely tend to grow faster than companies with employees working in an office. |
| 1. LIK_18O_ | Companies with employees working in an office tend to grow faster than companies with employees working remotely. |
| 1. LIK_19R_ | Companies with employees working remotely tend to have lower costs of absenteeism than companies with employees working in an office. |
| 1. LIK_19O_ | Companies with employees working in an office tend to have lower costs of absenteeism than companies with employees working remotely. |

# Appendix C: Misinformation stimuli

## Pro-remote-work misinformation

**Remote work leads to more success than work in a traditional office**

By Thomas Perk | Published on November 15, 2020

The debate on pros and cons of remote and in-office work has gained more and more attention in the last few years. A recent international research study by the U.K. Work Research Institute (WRI) seems to finally bring clarity to this debate. Indeed, after analyzing over 300 large and medium-sized European companies, the WRI conclude in their working paper that companies with employees working remotely are more successful than companies with employees working in a traditional office.

Employees working remotely have greater autonomy and more flexibility, as they can decide when to work. “Remote workers do not have to work from 9 am to 5 pm as their counterparts in a traditional office. They can choose the time of the day they are more focused and productive”, says lead researcher Prof. T. Scott from the WRI. Remote workers also face much fewer interruptions from their colleagues during the work than their counterparts in a traditional office environment. All these factors contribute to an increase in their own and thus also their company’s productivity in the long term. Additionally, this also brings significant savings to the companies with employees working remotely in terms of costs caused by low productivity of employees.

The study also showed that employees working remotely are happier, less stressed out and healthier than office workers. This does not only reduce companies’ costs of absenteeism but it also leads to higher productivity and efficiency of the companies with employees working remotely. “Remote workers also have higher morale and enjoy their job responsibilities more, which makes them more engaged and committed to the company in comparison to employees working in an office”, says Prof. T. Scott. Remote workers are thus less likely to change their job, which results in the reduction of employee turnover rate in the company. It is estimated that employee turnover costs the companies between 40% and 75% of employee’s annual salary on average. A lower employee turnover rate thus saves the companies with employees working remotely a significant amount of money in comparison to the companies with employees working in an office. A lower employee turnover rate also results in a higher productivity and efficiency of the companies with employees working remotely. Letting employees work remotely also reduces the overhead expenditures. Companies with employees working remotely need no or a lot less office space than companies with employees working in an office and they also save on office equipment, power bills, refreshment expenditure, etc.

Overall, the study suggests that greater autonomy, more flexibility, better health as well as higher morale, more engagement and more commitment to the company of remote workers make the companies with employees working remotely more productive, efficient, and thus more successful in comparison to the companies with employees working in a traditional office. This is a strong argument for the companies to switch to remote work if they have not done so yet due to the current situation with the COVID-19 pandemic.

**
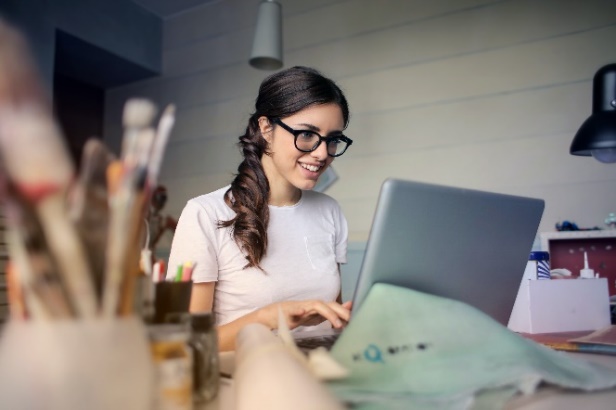

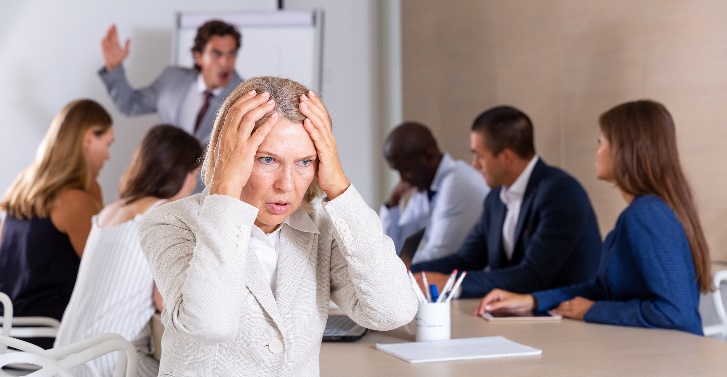
**

*Remote workers are more engaged and productive Office workers are more stressed*

## Pro-in-office-work misinformation

**Work in a traditional office leads to more success than remote work**

By Thomas Perk | Published on November 15, 2020

The debate on pros and cons of remote and in-office work has gained more and more attention in the last few years**.** A recent international research study by the U.K. Work Research Institute (WRI) seems to finally bring clarity to this debate. Indeed, after analyzing over 300 large and medium-sized European companies, the WRI conclude in their working paper that companies with employees working in a traditional office are more successful than companies with employees working remotely.

Employees working in a traditional office receive more guidance and support from their supervisors and colleagues than their counterparts working remotely. When they have a problem, they can easily call in the relevant people to solve it. When they have a significant decision to make, they can gather their team to discuss it. Employees working remotely often have to deal with such problems and decisions alone. Another important factor is teamwork. “Office workers spend time with their team, strive towards one goal, search together for the best strategies to carry their work forwards. There is also healthy competitiveness among colleagues. Remote workers miss both the spirit of teamwork and the competitive environment”, says lead researcher Prof. T. Scott from the WRI. Employees working in an office also face much fewer interruptions during the work than their counterparts working remotely, who get easily distracted by household, family, or surfing on internet. All these factors contribute to an increase in their own and thus also their company’s productivity in the long term. Additionally, this also brings significant savings to the companies with employees working in an office in terms of costs caused by low productivity of employees.

The study also showed that employees working remotely often cannot switch off from work, are unable to separate work and free time, are lonely and have difficulties to collaborate. This is not the case for employees working in a traditional office with 9 am to 5 pm schedules and easy access to their colleagues. Thus, office workers are happier, less stressed out and healthier than remote workers. This does not only reduce companies’ costs of absenteeism but it also leads to higher productivity and efficiency of the companies with employees working in an office. “Office workers also have higher morale and enjoy their job responsibilities more, which makes them more engaged and committed to the company in comparison to employees working remotely”, says Prof. T. Scott. Office workers are thus less likely to change their job, which results in the reduction of employee turnover rate in the company. It is estimated that employee turnover costs the companies between 40% and 75% of employee’s annual salary on average. A lower employee turnover rate thus saves the companies with employees working in a traditional office a significant amount of money in comparison to the companies with employees working remotely. A lower employee turnover rate also results in a higher productivity and efficiency of the companies with employees working in an office.

Overall, the study suggests that more guidance and support, better health as well as higher morale, more commitment to the company and more engagement of office workers make the companies with employees working in a traditional office more productive, efficient, and thus more successful in comparison to the companies with employees working remotely. This is a strong argument for the companies to stick to work in a traditional office or return to it if they were forced to temporarily switch to remote work due to the COVID-19 pandemic.


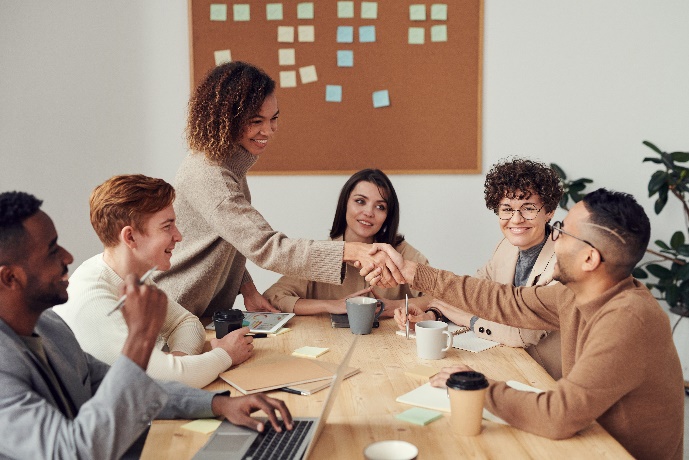

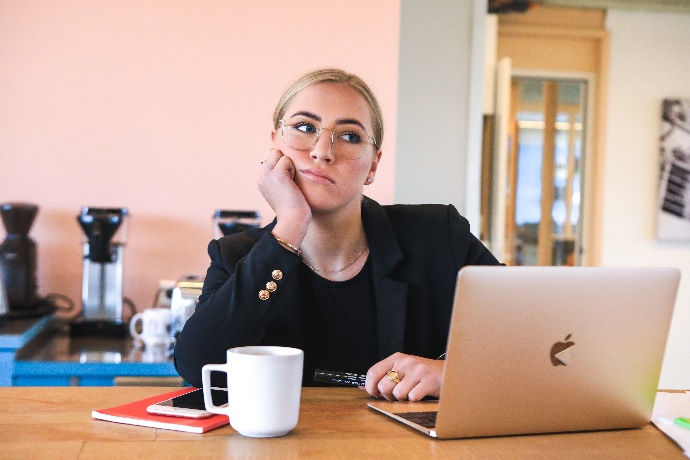


*Office workers benefit from working in a team Remote workers miss the spirit of teamwork*

# Appendix D: Debiasing treatment conditions

## Counter-speech – arguments supporting an opposite claim (CS_opp_)

You already know that the article you read at the beginning of the survey had actually been deleted from the blog due to the rejection of the research paper referred to in the article. The claim in the article that remote work would lead to an increase in companies’ productivity and efficiency is therefore not substantiated. There are actually many arguments suggesting the opposite, i.e. that **work in a traditional office increases companies’ productivity and efficiency** compared to remote work.

For example:

1. According to [Gensler’s U.S. Workplace Survey 2020](https://www.gensler.com/gri/us-workplace-survey-2020-summer-fall), over one half of the respondents say that they are **more productive** when working in an office.
2. Working in a traditional office enables **better collaboration**. Actually, according to [Gensler’s France Workplace Survey 2020](https://www.gensler.com/gri/france-workplace-survey-2020-en), the majority of workers believe that it is easier to collaborate if everyone works from the office. Easier collaboration, in turn, increases employees’ and thereby the company’s efficiency.
3. According to [Judith Olson’s research](https://www.researchgate.net/publication/220878926_Remote_and_alone_Coping_with_being_the_remote_member_on_the_team#fullTextFileContent), a distance-work expert and professor at the University of California, and her colleagues, working in an office creates a **routine to help structure a person’s workday**, while remote workers do not have a clean separation of work from private life. A better structure of workday results in improved productivity and efficiency of the workers and the companies.

Spend some time thinking about these arguments and then try to think of other arguments suggesting that **work in a traditional office increases companies’ productivity and efficiency** compared to remote work.


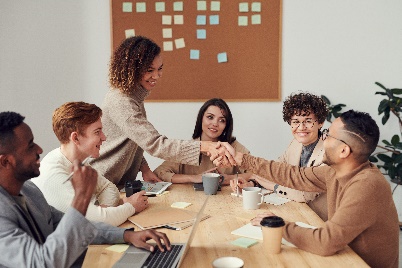


*Office workers can better collaborate*

## Counter-speech – arguments invalidating the specific claim (CS_inv_)

You already know that the article you read at the beginning of the survey had actually been deleted from the blog due to the rejection of the research paper referred to in the article. The claim in the article that remote work would lead to an increase in companies’ productivity and efficiency is therefore not substantiated. There are actually many arguments suggesting that **remote work does not increase companies’ productivity and efficiency** compared to work in a traditional office.

For example:

1. According to [Buffer´s 2020 State of Remote Work report](https://buffer.com/state-of-remote-work/2020), remote workers **struggle with unplugging after work and loneliness**. These can lead to depressions and decreased productivity.
2. According to [Judith Olson’s research](https://www.researchgate.net/publication/267257826_Bridging_Distance_Empirical_Studies_of_Distributed_Teams), a distance-work expert and professor at the University of California, and her colleagues, remote work can lead to **frustrating delays in getting collaborative work done**. These delays result in decreased efficiency.
3. [2018 Workplace Trends and Virgin Pulse survey](https://hbr.org/2018/11/survey-remote-workers-are-more-disengaged-and-more-likely-to-quit) found out that remote workers are far **less engaged in their work and show less long-term commitment to their employer**. This harms companies’ productivity and efficiency.

Spend some time thinking about these arguments and then try to think of other arguments suggesting that **remote work does not increase companies**’ **productivity and efficiency** compared to work in a traditional office.


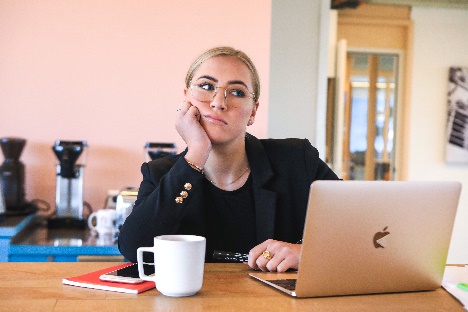


*Remote workers are not engaged in their work*

## Awareness-training – medical hoax (AT_hoax_)

You already know that the article you read at the beginning of the survey had actually been deleted from the blog. Therefore, **the article** **should theoretically have no influence on your opinion** regarding the impact of remote and in-office work on companies’ productivity and efficiency. Nevertheless, the opposite is likely to be true. It appears that people often insist on opinions or hypotheses, even after they have found that they are based on wrong, incomplete or misleading information. This **irrational behavior, called** "***belief perseverance”***, is responsible for the sustainable perseverance of fake news in the minds of many people.

The effect of belief perseverance can be explained on **the most damaging medical hoax of the last 100 years**. In 1998, a paper stating that the measles-mumps-rubella vaccine causes autism was published and triggered a lot of debate over the safety of the vaccine. As a consequence, the vaccination compliance dropped sharply in the years after the publication. Later, the paper was officially labeled fraud and fully retracted. Numerous well-controlled follow-up studies found no link between the vaccine and autism. If people were to act rationally, they would have to completely disregard the fraudulent information about the link between the vaccine and autism. Nevertheless, many parents still refuse to vaccinate their children and members of anti-vaccine movements still promote conspiracy theories linking the vaccination and autism.

It is important that people create and change their opinion and make decisions freely without being manipulated by misinformation. Therefore, **you should be aware of this trap in form of “belief perseverance” whenever you come across misinformation or fake news**.

## Awareness-training – hypothetical real-life situation (AT_hyp_)

You already know that the article you read at the beginning of the survey had actually been deleted from the blog. Therefore, **the article** **should theoretically have no influence on your opinion** regarding the impact of remote and in-office work on companies’ productivity and efficiency. Nevertheless, the opposite is likely to be true. It appears that people often insist on opinions or hypotheses, even after they have found that they are based on wrong, incomplete, or misleading information. This **irrational behavior, called** "***belief perseverance”***, is responsible for the sustainable perseverance of fake news in the minds of many people.

The effect of belief perseverance can be further illustrated by the following situation: Emily tells Oliver that the new fellow student Paul is not particularly clever and advises him against working in a group with Paul. After few days, Oliver finds out that there was a misunderstanding and that Emily did not speak about Paul but about Tim. If Oliver was to act rationally, he would now be completely free of prejudices against Paul, since his original opinion about Paul turned out to be based on wrong information. Nevertheless, due to belief perseverance, Oliver continues to avoid working with Paul, even though he knows Emily has been talking about someone else.

It is important that people create and change their opinion and make decisions freely without being manipulated by misinformation. Therefore, **you should be aware of this trap in form of “belief perseverance” whenever you come across misinformation or fake news**.

## Control group (CG)

We would like to learn about **your security habits**. Please answer the following questions.

|  | Yes | Maybe | No |
| --- | --- | --- | --- |
| Have you ever kept your passwords in your cell phone, computer, or your email? |  |  |  |
| Do you ever write your passwords in planners, paper, or sticky notes? |  |  |  |
| Does anyone else in your family have or use your passwords? |  |  |  |
| Have you ever shared any of your passwords with a colleague or a friend? |  |  |  |
| Have you ever shared your workplace passwords with a third-party? |  |  |  |
| Would you consider selling your workplace passwords to a third-party? |  |  |  |
| Do you sometimes reuse a password for multiple sites or applications? |  |  |  |
| Do you change your passwords at least 1 x per year? |  |  |  |
| Does any of your passwords contain any personal information? |  |  |  |
| Do you regularly make backups of files to another saving device or use any cloud solutions (e.g. OneDrive, Google drive, Dropbox)? |  |  |  |
| Do you often receive spam emails? |  |  |  |
| Do you ever open spam emails? |  |  |  |
| Do you think that you have ever been exposed to a phishing attack (fraudulent attempt to obtain sensitive information or data)? |  |  |  |
| Have you ever had a computer virus? |  |  |  |
